# Identification of chicken-derived antibodies targeting the *Candida albicans* Als3 protein

**DOI:** 10.1007/s00253-025-13469-3

**Published:** 2025-04-08

**Authors:** Chi-Hsin Lee, Chao-Jung Wu, Fang-Yi Yen, Jia-Yun Chiang, Ting-Jing Shen, Sy-Jye Leu, Chuang-Rung Chang, Hsiu-Jung Lo, Bor-Yu Tsai, Yan-Chiao Mao, Valencia Andriani, Priskila Cherisca Thenaka, Wei-Chu Wang, Yu-Pin Chao, Yi-Yuan Yang

**Affiliations:** 1 School of Medical Laboratory Science and Biotechnology, College of Medical Science and Technology, Taipei Medical University, No. 301, Yuantong Rd., Zhonghe Dist., New Taipei City, 235 Taiwan; 2 Ph.D. Program in Medical Biotechnology, College of Medical Science and Technology, Taipei Medical University Inc., Taipei, 110301 Taiwan; 3 Core Laboratory of Antibody Generation and Research, Taipei Medical University, Taipei, 110301 Taiwan; 4 Department of Microbiology and Immunology, School of Medicine, College of Medicine, Taipei Medical University, Taipei, 110301 Taiwan; 5 Institute of Biotechnology, National Tsing Hua University, Hsinchu, 300044 Taiwan; 6 National Institute of Infectious Diseases and Vaccinology, National Health Research Institutes, Miaoli, 350401 Taiwan; 7 Navi Bio-Therapeutics Inc., Taipei, 10351 Taiwan; 8 Division of Clinical Toxicology, Department of Emergency Medicine, Taichung Veterans General Hospital, Taichung, 407219 Taiwan; 9 iReal Biotechnology Inc., Hsinchu, 30060 Taiwan

**Keywords:** *Candida albicans*, Agglutinin-like sequence protein 3 (Als3), Chicken IgY, Phage display, Single-chain antibody (scFv)

## Abstract

**Abstract:**

*Candida albicans* is a major opportunistic pathogen, responsible for nearly half of clinical candidemia cases. The rising prevalence of azole-resistant *Candida* species represents a significant clinical challenge, underscoring the urgent need for alternative therapeutic strategies. Monoclonal antibody-based therapies have emerged as a promising and cost-effective approach to combating *Candida* infections. Agglutinin-like sequence protein 3 (Als3), a key cell surface protein of *C. albicans*, plays a pivotal role in adherence and biofilm formation, both of which are essential for its pathogenesis. In this study, recombinant Als3 protein was purified and utilized to immunize chickens, resulting in the production of Als3-specific immunoglobulin Y (IgY) antibodies. Two single-chain variable fragment (scFv) antibody libraries were subsequently constructed using phage display technology, yielding transformant counts of 5.3 × 10^7^ and 2.8 × 10^7^, respectively. Phage-based enzyme-linked immunosorbent assay (ELISA) revealed enhanced signals following bio-panning, enabling the identification and sequence validation of three scFv antibodies. These scFv antibodies exhibited strong binding activities to Als3, as confirmed through ELISA and western blot analyses. Binding affinities were determined to be ~ 10⁻⁸ M via serial titration ELISA and competitive ELISA. Additionally, the selected scFv antibodies specifically recognized endogenous Als3 protein in *C. albicans*, as demonstrated by western blot and cell-based ELISA assays. In conclusion, this study successfully generated and characterized high-affinity scFv antibodies targeting Als3, which exhibited exceptional specificity and binding activity. These findings highlight their potential as promising immunotherapeutic candidates for the treatment of *C. albicans* infections.

**Key points:**

• *The Als3 protein of C. albicans is a critical biomarker and therapeutic target*

• *Chicken-derived scFv antibodies against Als3 were developed via phage display*

• *The scFv antibodies showed strong binding to endogenous Als3 in C. albicans*

## Introduction

*Candida* infections are persistent, challenging to treat, and associated with significant morbidity and mortality, even with the availability of advanced antifungal therapies. Globally, *Candida* species rank as the third most common cause of infections in intensive care units (ICUs) (Paramythiotou et al. 2014; Tabah et al. 2012). They are also the fourth leading cause of hospital-acquired infections and the third most common pathogens implicated in bloodstream infections, underscoring their substantial clinical relevance (Alkharashi et al. 2019; Li et al. 2016). The diagnosis of invasive candidiasis remains particularly complex due to its nonspecific clinical manifestations and the low sensitivity of blood cultures, which, despite being the gold standard for detecting candidemia, achieve a sensitivity of only approximately 40% when the fungus infiltrates the bloodstream and disseminates to internal organs, causing invasive infections (Berenguer et al. 1993; Morace and Borghi 2010). Among *Candida* species, *Candida albicans* is the predominant fungal pathogen responsible for human diseases (Guinea 2014; Yapar 2014). While Amphotericin B (AmB) has historically been considered the gold standard for antifungal treatment, its severe toxicity significantly limits its clinical applicability (Deray 2002). Furthermore, the extensive and prolonged use of azole antifungal agents has accelerated the emergence of multidrug resistance (MDR) in *Candida* species (Pfaller 2012; Wisplinghoff et al. 2004). Of particular concern, the prevalence of fluconazole-resistant *Candida* strains has increased dramatically over the past two decades, presenting significant challenges to effective therapeutic management (Danby et al. 2012; Marchaim et al. 2012; Pinto et al. 2008; Sekhavat et al. 2011). Infections caused by *C. albicans* are intricately associated with biofilm formation, a process characterized by surface attachment, which plays a critical role in the pathogens virulence and persistence (Sudbery 2011). Notably, biofilm-forming *C. albicans* exhibits significantly greater tolerance to antimycotics compared to its planktonic counterparts, frequently leading to therapeutic failure and heightened morbidity and mortality rates (Ning et al. 2015; Pereira et al. 2021).

The *C. albicans* agglutinin-like sequence (Als) protein family, particularly Als3, has been identified as a key player in promoting filamentation and biofilm formation in *C. albicans* (Roudbarmohammadi et al. 2016; Zhao et al. 2004). These processes are essential to the pathogens virulence, aiding in its adhesion to host surfaces, tissue invasion, and establishment of chronic infections (Cota and Hoyer 2015; Zhao et al. 2004). Als3, a cell surface glycoprotein, exhibits a broad range of adhesion capabilities, interacting with host cell-specific receptors such as cadherins, collagens, and fibrinogen, thereby significantly contributing to *C. albicans* pathogenicity (Cota and Hoyer 2015; Hoyer and Cota 2016; Liu and Filler 2011; Zhao et al. 2004; Zhu et al. 2012). Additionally, Als3 interacts with the epidermal growth factor receptor (EGFR), facilitating the endocytosis of *C. albicans* into host cells and exacerbating host cell damage by acquiring iron from host ferritin (Almeida et al. 2008; Zhu et al. 2012). The upregulation of Als3 expression in *C. albicans* has been strongly correlated with increased biofilm formation, underscoring its pivotal role as both a biomarker and a major contributor to biofilm development (Sui et al. 2017). Furthermore, Als3 has emerged as a promising target for immunotherapeutic interventions against *Candida* infections. Notably, passive immunotherapy using anti-Als3 antibodies has been proposed as an innovative approach to combat infections caused by *Candida* species (Singh et al. 2019). These findings emphasize the potential of developing Als3-specific antibodies as a novel therapeutic strategy, offering significant promise for overcoming the challenges associated with *C. albicans* infections and improving antifungal treatment outcomes.

Immunotherapy utilizing antibodies has demonstrated remarkable efficacy across a broad range of targets, including cancers, autoimmune disorders, and infectious diseases (Wang et al. 2022). A key milestone in this field was achieved by Smith (1985) and Huse et al. (1989), who revolutionized antibody development with the invention and application of phage display technology, significantly expanding the possibilities for generating monoclonal antibodies. Since then, phage display technology has become a versatile and cost-effective platform for selecting highly specific antibodies against a variety of targets, including cancers, viruses, and bacterial pathogens (Guliy et al. 2024). This innovative approach continues to play a pivotal role in advancing immunotherapeutic strategies and has been successfully applied in diverse species, including chickens, which offer a cost-efficient and readily available alternative for monoclonal antibody production (Yang et al. 2024). In addition to intact IgG antibodies, recombinant single-chain variable fragment (scFv) antibodies are gaining significant recognition in the biotechnology market due to their superior tissue penetration, smaller size, and ease of production via phage display technology. These scFv antibodies are increasingly utilized in both diagnostic and therapeutic applications, underscoring their growing importance in expanding the scope and versatility of immunotherapy. Given their cost-effectiveness and accessibility, chickens provide a practical and widely available host for constructing scFv antibody libraries (Park et al. 2005). These libraries enable the efficient screening of high-affinity binders targeting various pathogens, thereby enhancing their potential applications in diagnostics and therapeutics.

Building on the findings of previous studies, we propose that Als3 in *C. albicans* represents a promising target for the development of novel diagnostic and therapeutic applications. In this study, we aim to generate specific antibodies against Als3 using a dual approach: the production of anti-Als3 polyclonal IgY antibodies through chicken immunization and the generation of monoclonal scFv antibodies via phage display technology. The binding affinities and specificities of these antibodies will be rigorously assessed using ELISA and western blot analyses. We anticipate that the anti-Als3 polyclonal IgY and monoclonal scFv antibodies developed in this study could serve as essential tools for the future design of diagnostic assays and therapeutic strategies targeting *C. albicans* infections.

## Materials and methods

### Chicken immunization

The His-tagged Als3 proteins, expressed in the *Saccharomyces cerevisiae* W303 (ATCC 208352) model and purified using Ni^2+^ Sepharose resin, were kindly provided by Professor Chuang-Rung Chang from the College of Life Science and Medicine, National Tsing Hua University, Hsinchu, Taiwan (Huang et al. 2024). Female brown ISA chickens (*Gallus gallus domesticus*) were initially immunized with 50 µg of purified Als3 emulsified in Complete Freund’s Adjuvant, administered intramuscularly at multiple sites on the thighs. Subsequent immunizations with Als3 mixed with Incomplete Freund’s Adjuvant were performed at 7-day intervals. Polyclonal IgY antibodies were purified using the dextran sulfate method (Akita and Nakai 1993), and ELISA was employed to assess the humoral immune response.

### Construction and bio-panning of scFv antibody libraries

The antibody libraries were constructed using phage display technology, as previously described (Andris-Widhopf et al. 2000). Briefly, spleens were homogenized in Trizol solution, and 20 µg of total RNA was reverse transcribed into first-strand cDNA using the SuperScript RT Kit (Invitrogen, Waltham, MA, USA). cDNA templates were then amplified through a two-step PCR process. In the first round, immunoglobulin light-chain variable region (*V*_L_) and heavy-chain variable region (*V*_H_) fragments were amplified using specific chicken primers. In the second round, full-length *scFv* genes with either a short or long peptide linker were generated. The scFv and pComb3X vector DNA (Barbas et al. 1991) were digested with the *Sfi*I restriction enzyme (New England Biolabs, Ipswich, CA, USA), and the ligated full-length *scFv* gene was cloned into the pComb3X vector to produce recombinant phagemid DNA. These recombinant phagemids were then transformed into *Escherichia coli* ER2738 (New England Biolabs, Ipswich, CA, USA) by electroporation (MicroPulser, BioRad, Hercules, CA, USA). Recombinant phages were produced by infecting the transformed *E. coli* with wild-type VCS-M13 helper phage (Stratagene, La Jolla, CA, USA). After overnight culture, the phages in the supernatant were precipitated using 4% polyethylene glycol 8000 and 3% NaCl. Following centrifugation, the phages were resuspended in PBS containing 1% bovine serum albumin (BSA) and 20% glycerol then stored at − 20 °C.

Recombinant phages displaying the scFv library, at a concentration of 10^11^–10^12^ plaque-forming units (PFU), were incubated in wells coated with Als3 protein (0.5 µg/well). After incubation, unbound phages were removed by washing with PBST (PBS containing 0.05% Tween 20). Bound phages were eluted with 0.1 M glycine–HCl (pH 2.2), neutralized with 2 M Tris-base, and used to infect *E. coli* ER2738. A portion of the infected bacteria was plated to calculate the eluted phage titer by colony-forming units (CFU), while the remaining bacteria were used to amplify recombinant phages and collected as previously described. This process was repeated for additional rounds of selection under identical conditions.

### Protein expression and purification of scFv antibodies

After bio-panning, recombinant phagemid DNA was transformed into *E. coli* TOP10F' (Invitrogen, Waltham, MA, USA). Individual clones were randomly selected, cultured overnight, and diluted 1:100 in super broth (3% tryptone, 2% yeast extract and 1% 3-(*N*-morpholino)propanesulfonic acid (MOPS), pH 7.0) containing 20 mM MgCl₂ and 50 µg/mL ampicillin. The cultures were incubated for 8 h, after which, the protein expression was induced by adding 1 mM isopropyl-β-d-thiogalactopyranoside (IPTG) and continuing incubation overnight. *E. coli* cells were collected by centrifugation, resuspended in His-binding buffer (20 mM sodium phosphate, 0.5 M NaCl, 20 mM imidazole, pH 7.4), and lysed via sonication. The scFv antibodies were subsequently purified using Ni^2^⁺ Sepharose resin (Cytiva, Global Life Sciences Solutions USA LLC, Wilmington, DE, USA) following the manufacturers instructions.

### Sequence analysis

Nucleotide sequences of scFv-expressing clones were determined using the ompseq (5′-AAGACAGCTATCGCGATTGCAGTG-3′) primer, and their putative amino acid sequence *V*_L_ and *V*_H_ were aligned with those of the chicken immunoglobulin germ line (Andris-Widhopf et al. 2000). The sequences of selected three scFv clones, cAls3-S1 (PV056497), cAls3-L1 (PV056498), and cAls3-L7 (PV056499) were submitted to NCBI GenBank.

### Electrophoresis and western blot analysis

Sodium dodecyl sulfate polyacrylamide gel electrophoresis (SDS-PAGE) was performed as previously described, using a 4% stacking gel and a 10% or 12% resolving gel (Bio-Rad Laboratories Inc., CA, USA) (Laemmli 1970). After separation by 10% SDS-PAGE, the Als3 protein or total cell lysates were transferred onto polyvinylidene difluoride (PVDF) membranes and blocked with PBS containing 5% skim milk for 1 h at room temperature (25 °C). All antibodies were diluted in PBS containing 5% skim milk. His-tagged Als3 protein was detected using rabbit anti-His antibody (1:5,000; iReal Biotechnology Inc., Hsinchu, Taiwan) with a 1 h incubation at 25 °C. Following three washes with PBST, horseradish peroxidase (HRP)-conjugated goat anti-rabbit IgG antibody (1:10,000; Jackson ImmunoResearch, West Grove, PA, USA) was applied and incubated for an additional hour. Antibody binding was visualized using diaminobenzidine (DAB) substrate after three further PBST washes. To assess the immune response, purified IgY antibodies (1:5000; stock concentration, 10 mg/mL) from pre-immunization and the sixth immunization were applied and detected with HRP-conjugated donkey anti-chicken IgY antibody (1:10,000; Jackson ImmunoResearch, West Grove, PA, USA). Additionally, *E. coli* lysates expressing scFv antibodies were diluted 1:10 and detected using mouse anti-HA tag antibody (iReal Biotechnology Inc., Hsinchu, Taiwan), followed by HRP-conjugated rabbit anti-mouse IgG antibody (Jackson ImmunoResearch, West Grove, PA, USA). All steps, including blocking, washing, incubation, and color development, were performed following the described protocol.

### Enzyme-linked immunosorbent assay (ELISA) and competitive ELISA

Als3 and BSA proteins (0.5 µg/well) were immobilized on ELISA plates and incubated at 37 °C for 1 h. The plates were then blocked with PBS containing 5% skim milk for an additional hour at 37 °C. Purified IgY antibodies, derived from either pre-immunization or the sixth immunization, were serially diluted in two-fold (from 1:500 to 1:256,000) and added to the wells, followed by incubation for 1 h at 37 °C. After six washes with PBST, HRP-conjugated donkey anti-chicken IgY antibody was added and incubated at 37 °C for 1 h. Binding activities were detected by adding 3,3′,5,5′-tetramethylbenzidine (TMB) substrate, and the reaction was stopped using 1 N HCl. Absorbance was measured at 450 nm.

For the phage-based ELISA, 10^11^–10^12^ PFU of amplified phages from each bio-panning round were added to wells coated with Als3 proteins. Bound phages were detected using HRP-conjugated mouse anti-M13 antibody (Sino Biological, Beijing, China). To further validate specific binding activities, scFv antibodies were applied to wells coated with Als3 proteins and detected using a mouse anti-HA tag antibody followed by an HRP-conjugated rabbit anti-mouse IgG antibody.

In the competitive ELISA, Als3 proteins were serially diluted in two-fold (300 to 0.146 µg/mL) and mixed with an equal volume of scFv antibodies (cAls3-S1, 0.625 µg/mL; cAls3-L1, 0.156 µg/mL; cAls3-L7, 0.625 µg/mL). The mixtures were incubated at 37 °C for 1 h, then transferred to Als3-coated wells and incubated at 37 °C for an additional hour.

Blocking, washing, incubation, and color development steps were conducted under the same conditions as previously described. All ELISA results were expressed as the mean ± SD of two independent experiments, with each sample tested in duplicate wells. Data analysis was performed using GraphPad Prism 6 software (La Jolla, CA, USA).

### Preparation of *C. albicans* cell lysate and biofilm formation on 96-well plates for ELISA assay

The fungal strain used in this study, *C. albicans* (SC5314), was kindly provided by Dr. Hsiu-Jung Lo from the National Institute of Infectious Diseases and Vaccinology, National Health Research Institutes, Tainan, Taiwan. The strain was stored in 15% glycerol at − 80 °C. *C. albicans* cells were cultured overnight at 37 °C in Sabouraud Dextrose Broth (SDB), resuspended in PBS, and lysed by sonication.

Biofilm formation was conducted following a previously described protocol (Liu et al. 2021). Overnight cultured *C. albicans* cells were adjusted to a concentration of 1 × 10^6^ CFU/mL in tryptic soy broth (TSB). A 0.1 mL aliquot of the cell suspension was added to sterile, flat-bottom 96-well plates and incubated at 37 °C with 5% CO_2_ for 1.5 h under agitation at 50 rpm to facilitate initial adhesion. After incubation, the medium was removed, and the wells were washed twice with PBS to eliminate non-adherent cells. Fresh TSB medium was added to the wells, and the plates were incubated for 48 h to promote biofilm formation. Following biofilm development, the medium was removed, and methanol was added to the wells to fix the biofilms at 25 °C for 15 min. The wells were then washed twice with PBS and prepared for subsequent experiments, including cell-based ELISA for antibody binding assays.

## Results

### Chicken immunization for polyclonal IgY production

**Fig. 1:**
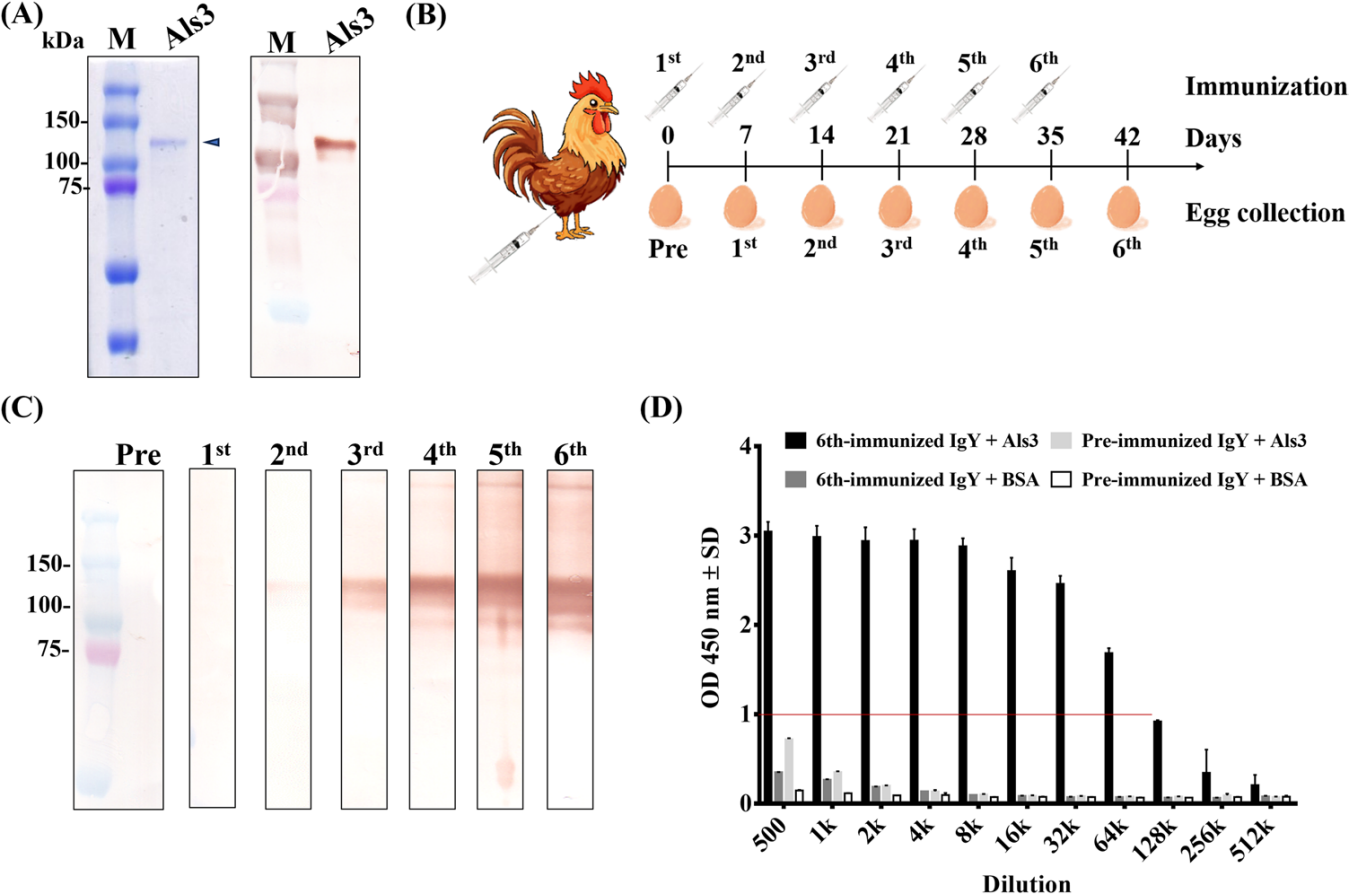
Characterization of the immune response to Als3 in chickens. **A** The purified Als3 protein was analyzed by Coomassie Brilliant Blue-stained SDS-PAGE, with the identity of Als3 confirmed by western blot using an anti-His antibody. **B** A schematic representation of the immunization process, illustrating the use of purified Als3 protein for immunization and the collection of eggs for antibody isolation. **C** The humoral immune response in chickens was monitored by western blot using purified IgY antibodies from pre- and post-immunization samples (first and sixth immunizations). **D** Purified IgY antibodies from pre- and sixth immunization were serially diluted (500 to 256,000 ×) and analyzed for their binding specificity to Als3 and BSA proteins using ELISA. ELISA data are presented as mean OD 450 nm ± SD from duplicate experiments

The purified Als3 protein was analyzed by SDS-PAGE, confirming its expected molecular weight and high purity. Its identity was further validated through western blot analysis using an anti-His antibody (Fig. 1A). The protein was emulsified with an adjuvant and administered intramuscularly to chickens at 7-day intervals to elicit a humoral immune response (Fig. 1B). IgY antibodies were extracted and purified from egg yolks collected before immunization (pre-immune) and after each of six immunizations (first to sixth) (data not shown). Western blot analysis of the IgY antibodies against the Als3 protein showed no detectable signal in pre-immune samples. A slight immune response was observed after the first immunization, with progressively stronger signals detected following subsequent immunizations (Fig. 1C). ELISA further confirmed the development of a robust humoral immune response, demonstrating sufficient antibody production for downstream analyses (Fig. 1D). IgY antibodies from the sixth immunization exhibited strong binding activity, with an OD value of 1.0 at a 1:64,000 dilution. In contrast, pre-immune IgY showed moderate binding activity (OD 0.4–0.8) at a 1:500 dilution, which declined to undetectable levels (OD < 0.2) with further dilution.

### Phage displaying scFv antibody library construction and bio-panning selection

Following immunization, the chicken was sacrificed, and the spleen was harvested for total RNA extraction. The extracted RNA served as a template for cDNA synthesis, enabling the amplification of *V*_L_ and *V*_H_. These fragments were subsequently assembled into full-length scFv using short or long linker and cloned into a phagemid vector. This strategy facilitated the construction of two distinct scFv antibody libraries, comprising 5.3 × 10^7^ and 2.8 × 10^7^ transformants, respectively, providing a robust and diverse repertoire for subsequent selection and characterization.

**Fig. 2:**
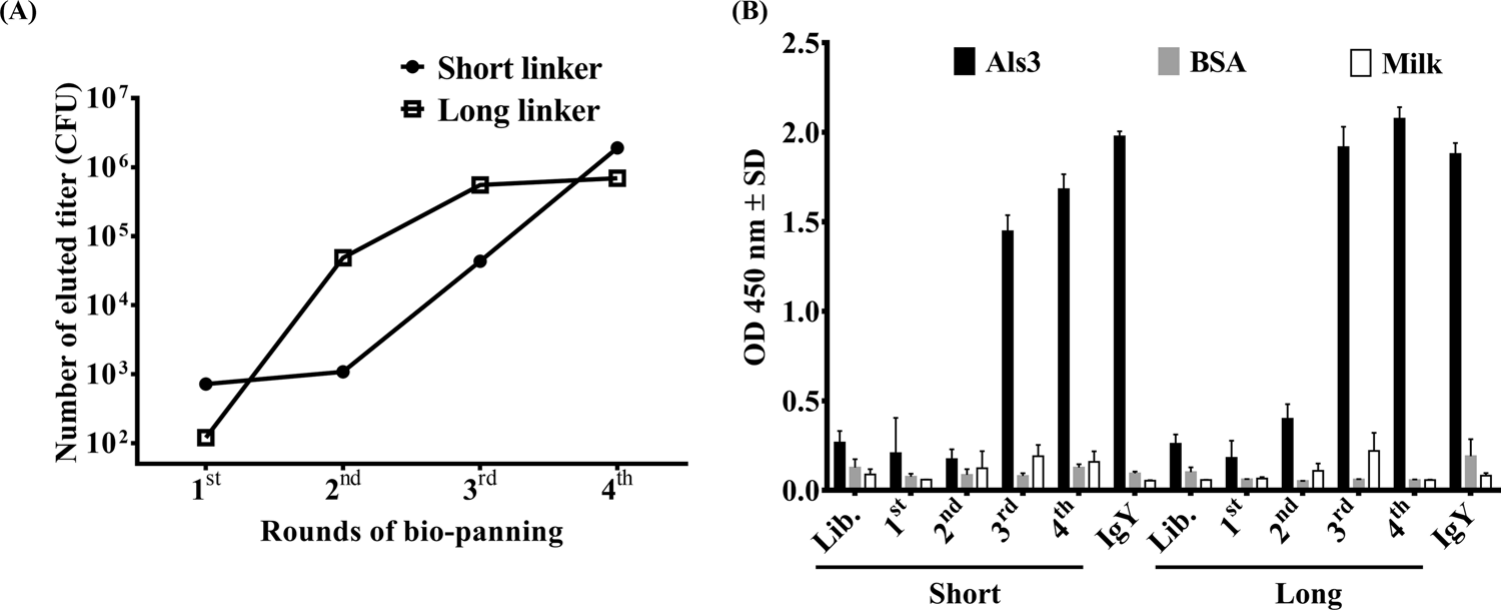
Eluted phage titers and specific binding activity of amplified phages during bio-panning. **A** Antibody libraries constructed with short and long linkers were subjected to bio-panning, and the titers of eluted phages were determined after each round of selection. **B** The binding abilities of amplified phages from each round of bio-panning to Als3 protein were evaluated using ELISA. Purified anti-Als3 IgY antibodies from the sixth immunization were used as a positive control. ELISA data are presented as mean OD 450 nm ± SD from duplicate experiments

During the bio-panning process, the eluted titers of both scFv libraries were closely monitored (Fig. 2A). In the first round, the eluted titer was close to 10^3^ CFU in the short-linker library and approximately 10^2^ CFU in the long-linker library. The short-linker library exhibited a gradual increase in eluted titers, reaching 10^6^ CFU by the third and fourth rounds. In contrast, the long-linker library showed a more pronounced increase in the second round compared to the short-linker library, eventually plateauing at approximately 5 × 10^5^ CFU during the third and fourth rounds. To validate the bio-panning process, a phage-based ELISA was performed (Fig. 2B). No detectable signals were observed in either library from the initial unselected phage preparations or from phages collected during the first and second rounds of bio-panning. However, increased signals were detected in both libraries during the third and fourth rounds, indicating successful enrichment of phages with specific binding affinities. These results confirm the effective isolation and selection of scFv antibodies with potential binding specificity, demonstrating the success of the bio-panning process.

### Analysis of scFv protein expression and binding activities

**Fig. 3:**
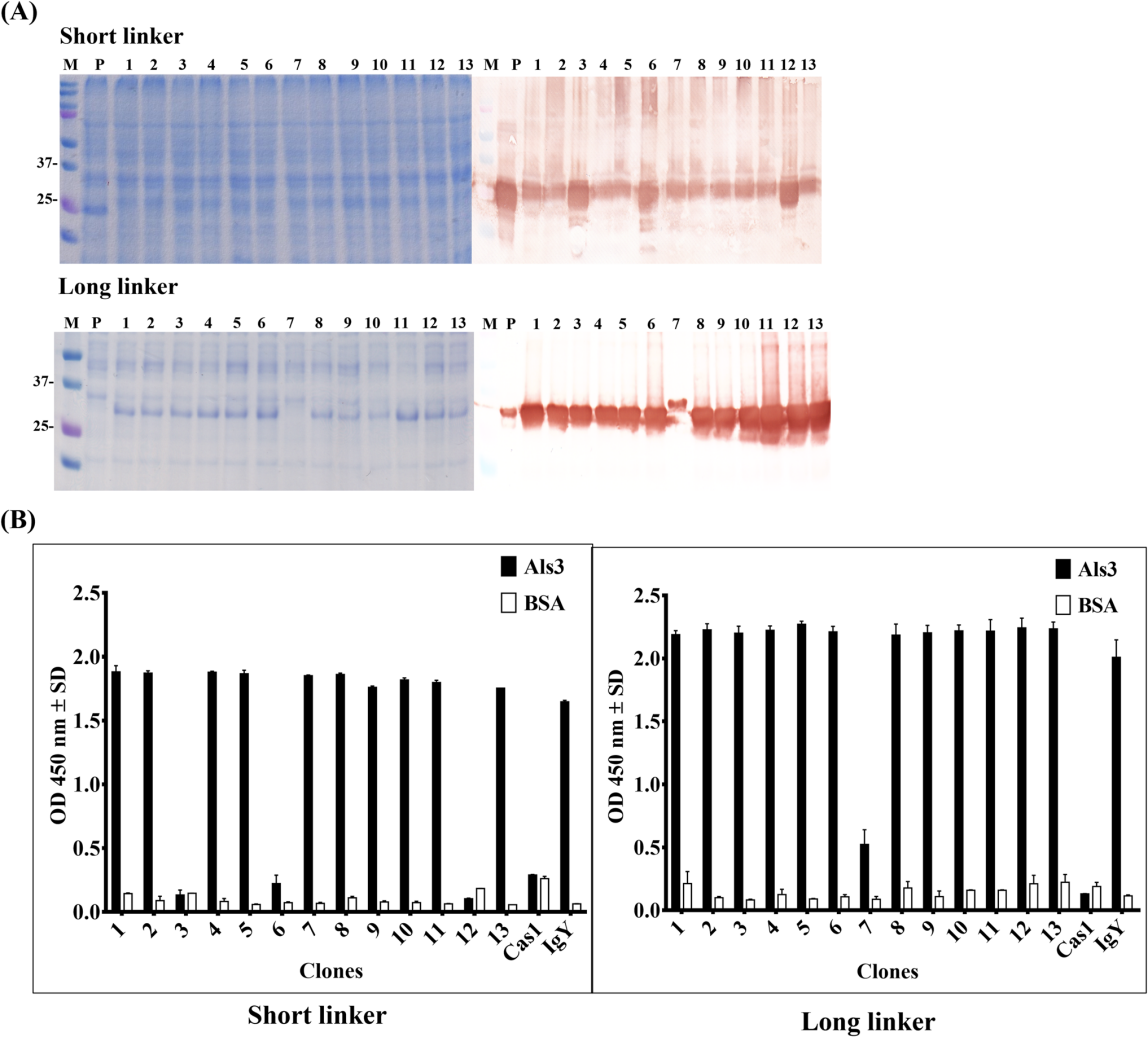
Binding analysis of randomly selected scFv antibodies following expression. **A** Thirteen randomly selected clones from the short-linker and long-linker libraries were induced with IPTG for scFv antibody expression. The expressed scFv antibodies were analyzed by Coomassie Brilliant Blue-stained SDS-PAGE and confirmed by western blot using an anti-HA antibody. Lane P represents the CaS1 scFv targeting *C. albicans* enolase. **B** The binding activities of the expressed scFv antibodies (without purification) to Als3 protein were assessed using ELISA. Purified IgY antibodies from the sixth immunization served as the positive control, while CaS1, targeting *C. albicans* enolase, served as the negative control. ELISA data are presented as mean OD 450 nm ± SD from duplicate experiments

Following the bio-panning process, thirteen randomly selected clones from each library were induced to express scFv antibodies via IPTG induction. The expressed scFv proteins were analyzed using SDS-PAGE and western blot to confirm their expression and molecular size (Fig. 3A). Consistent with the positive control, CaS1 (Lane P), which targets *C. albicans* enolase, all clones expressed scFv antibodies within the expected molecular weight range of 25–37 kDa. Some clones displayed similar binding patterns on western blot. The binding activities of these scFv antibodies to purified Als3 protein were evaluated using ELISA (Fig. 3B). In the short-linker library, most clones exhibited binding activity to the Als3 protein, except for clones S3, S6, and S12. Conversely, all clones from the long-linker library demonstrated binding activity to Als3. No binding signals were observed for CaS1.

To further characterize the clones, their expressed scFv antibodies were sequenced, and their amino acid sequences were analyzed for classification (Table 1). The results identified two distinct classifications within each library. In the short-linker library, the clones were categorized as cAls3-S1 (10/13; 76.9%) and cAls3-S3 (3/13; 23.1%). Similarly, in the long-linker library, the clones were classified as cAls3-L1 (12/13; 92.3%) and cAls3-L7 (1/13; 7.7%). These classifications revealed dominant clone types in each library, providing a basis for subsequent functional analyses. Additionally, the mutation rates of the selected clones amino acid sequences were compared with chicken germline sequences (Table 2). The analysis revealed higher mutation rates in the *V*_L_ and *V*_H_ complementary determining regions (CDRs), ranging from 23 to 62%, compared to the framework regions (FRs), which ranged from 6 to 15%. Notably, the CDR3 regions in both *V*_L_ and *V*_H_ exhibited the highest variability, with mutation rates ranging from 36 to 93%.

**Table 1:** Classification of scFv clones according to the identity of light- and heavy-chain variable regions

Short linker				Long linker			
Group (name)	Light chain	Heavy chain	Percentage	Group (name)	Light chain	Heavy chain	Percentage
Group 1 (cAls3-S1)	S1, S2, S4, S5, S7, S8, S9, S10, S11, S13	S1, S2, S4, S5, S7, S8, S9, S10, S11, S13	76.9%	Group 1 (cAls3-L1)	L1, L2, L3, L4, L5, L6, L8, L9, L10, L11, L12	L1, L2, L3, L4, L5, L6, L7, L8, L9, L10, L11, L12	92.3%
Group 2 (cAls3-S3)	S3, S6, S12	S3, S6, S12	23.1%	Group 2 (cAls3-L7)	L7	L7	7.7%

**Table 2:** Amino acid mutation rates of *V*_L_ and *V*_H_ after classification

Regions	CDR1	CDR2	CDR3	Total CDR	FR1	FR2	FR3	FR4	Total FR
*V*_L_	13–50%	0–43%	36–93%	23–57%	5–10%	5–10%	16–25%	0%	9–15%
*V*_H_	40–60%	35–41%	74–85%	55–62%	7–10%	7–14%	6–19%	0%	6–10%

### Analysis of selected clones against Als3 protein

**Fig. 4:**
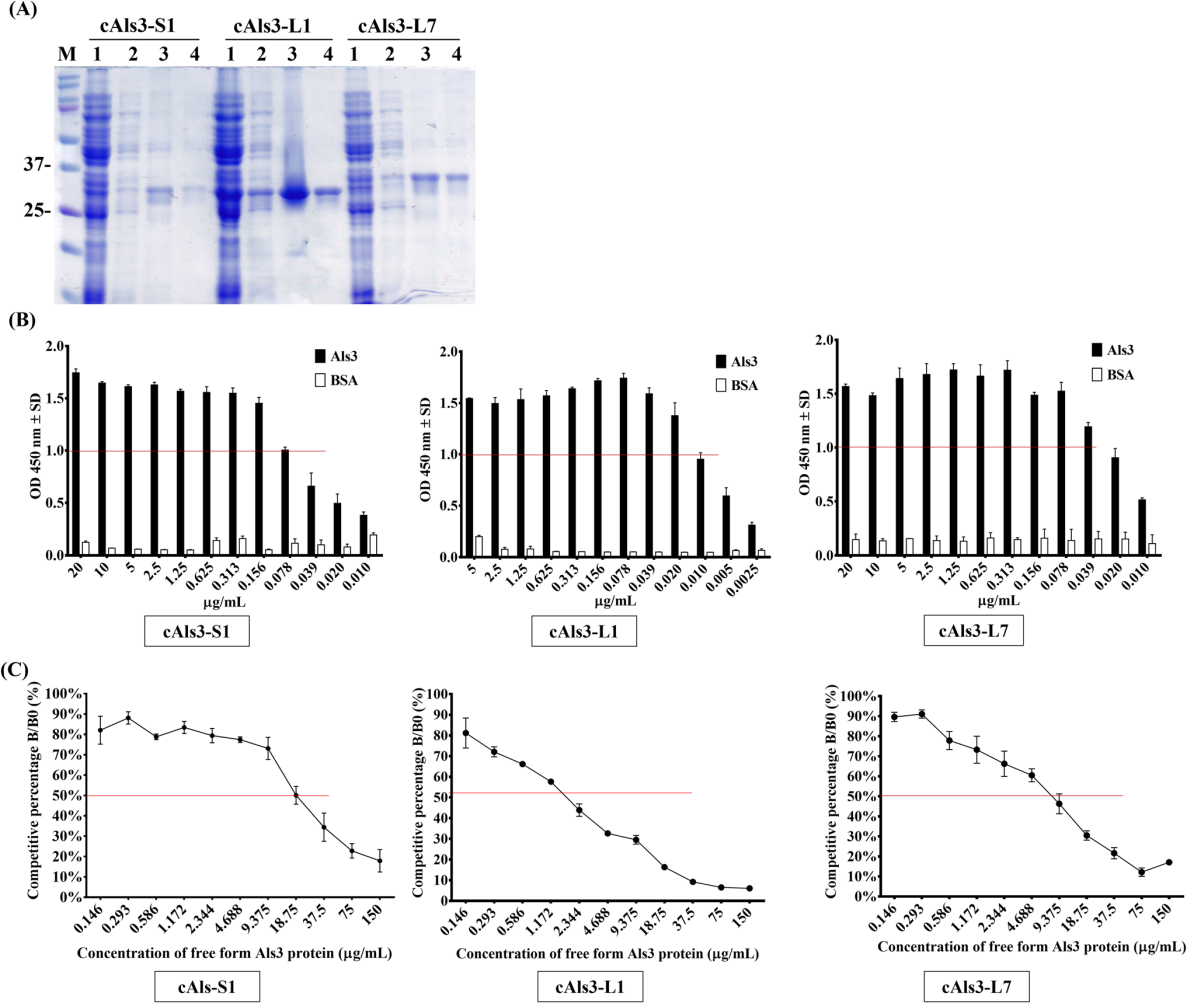
Binding affinity analysis of selected scFv antibodies against Als3 protein. **A** Three scFv antibodies were expressed following IPTG induction and purified using Ni^2^⁺ Sepharose affinity chromatography. Protein fractions were analyzed on SDS-PAGE. Lane 1: flow-through; Lane 2: wash fraction; Lane 3: elution fraction; Lane 4: Ni^2^⁺ Sepharose. **B** The binding activities of purified scFv antibodies were evaluated by performing a two-fold serial dilution ELISA. **C** Competitive ELISA was conducted to determine the binding affinities of the scFv antibodies. Purified scFv antibodies were incubated with varying concentrations of free Als3 protein before being added to immobilized Als3-coated wells. Binding inhibition was calculated as B/B₀, where B represents the bound scFv antibody in the presence of free Als3 protein, and B₀ represents the bound antibody in the absence of competition. ELISA data are expressed as mean OD 450 nm ± SD from duplicate experiments

Except for cAls3-S3, which showed no binding activity in ELISA (Fig. 3B), three selected scFv antibodies—cAls3-S1, cAls3-L1, and cAls3-L7—exhibited binding activity and were purified using Ni^2^⁺ Sepharose affinity chromatography. The purified antibodies were analyzed via 12% SDS-PAGE for further characterization (Fig. 4A), confirming successful purification in the elution fractions, with molecular weights ranging from approximately 25 to 37 kDa. To evaluate their binding activities to the Als3 protein, a titration assay was conducted using serial dilutions of the purified scFv antibodies (Fig. 4B). The results indicated that cAls3-S1 retained strong binding activity, with a gradual decline in signal intensity upon dilution, and detectable binding signals (OD > 1.0) at concentrations as low as 0.078 µg/mL. In comparison, cAls3-L1 and cAls3-L7 displayed stronger binding activities, maintaining OD values above 1.0 at lower concentrations of 0.02 µg/mL and 0.039 µg/mL, respectively. To further confirm their binding affinities, a competitive ELISA assay was performed at concentrations of 0.313 µg/mL, 0.078 µg/mL, and 0.156 µg/mL for cAls3-S1, cAls3-L1, and cAls3-L7, respectively (Fig. 4C). The results revealed varying binding affinities among the scFv antibodies, determined by their 50% inhibitory effects based on absorbance values. Specifically, cAls3-S1, cAls3-L1, and cAls3-L7 exhibited 50% inhibition effects at free-form Als3 protein concentrations of 18.75 µg/mL, 2.344 µg/mL, and 9.375 µg/mL, respectively. Overall, these findings suggest that cAls3-L1 possesses the highest binding affinity among the three tested scFv antibodies, highlighting its potential for further development in diagnostic and therapeutic applications.

### Binding assays of selected scFv antibodies against *C. albicans*

**Fig. 5:**
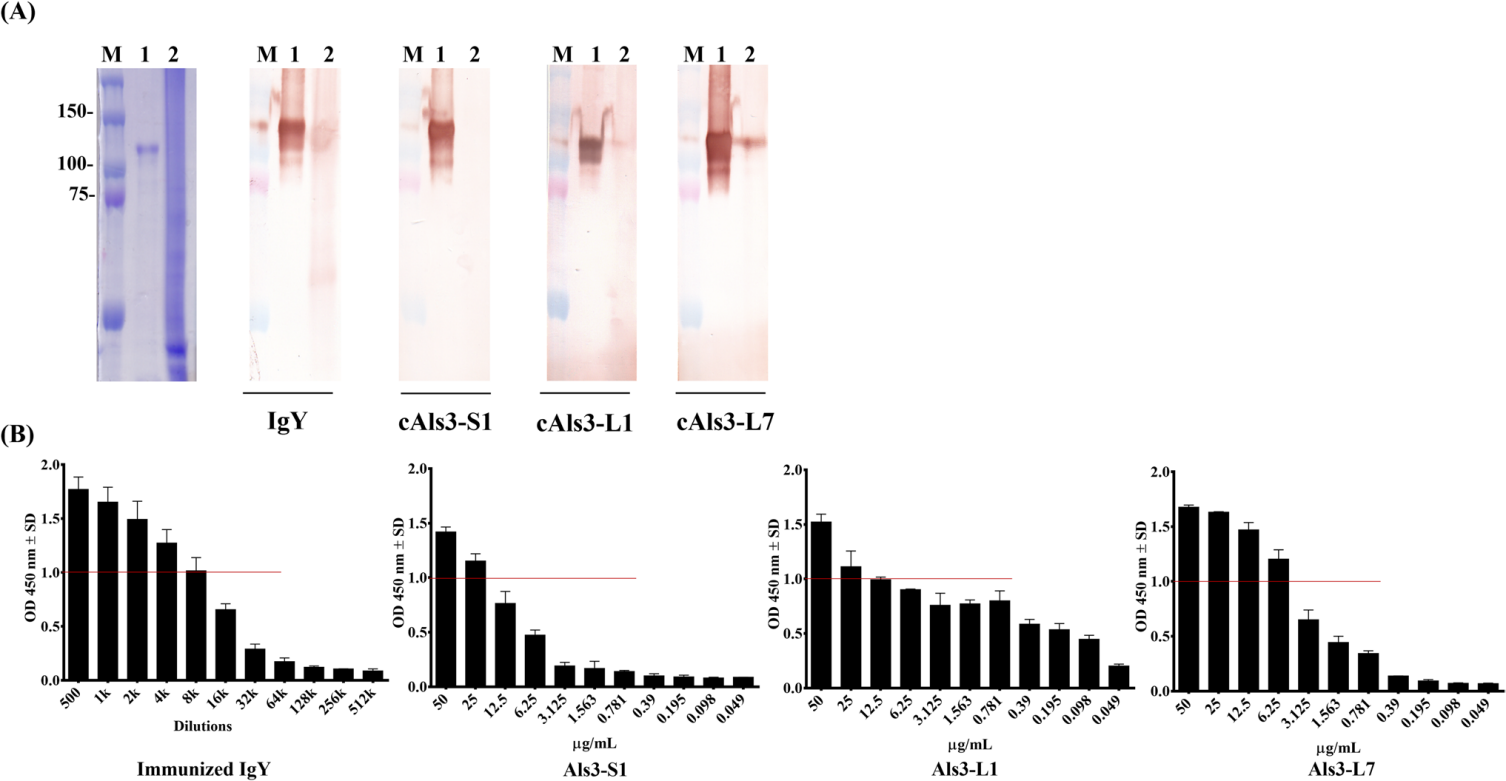
Binding assay of anti-Als3 antibodies against *C. albicans* recombinant Als3 protein and cell lysates, and biofilms. **A** Purified recombinant Als3 protein and *C. albicans* cell lysates were separated on SDS-PAGE, and transferred onto PVDF membranes. The membranes were incubated with purified IgY from Als3-immunized chicken or cell lysates containing scFv antibody (1:10 diluted). Lane 1 corresponds to recombinant Als3 protein, while Lane 2 corresponds to *C. albicans* cell lysates. **B** *C. albicans* overnight cultures were adjusted to 10^5^ CFU and added into sterile, flat-bottom 96-well plates. The cells were incubated at 37 °C with 5% CO_2_ for 1.5 h under gentle agitation to allow for adhesion. Non-adherent cells were removed, and TSB medium was added to promote biofilm formation, followed by incubation for 48 h. After incubation, the medium was removed, and biofilms were fixed with methanol. Purified antibodies, serially diluted in two-fold, were then added to assess their binding activity. ELISA data are presented as mean OD 450 nm ± SD from duplicate experiments

To further validate the binding specificity of the anti-Als3 scFv antibodies to *C. albicans*, PVDF membranes were prepared by immobilizing purified Als3 protein and *C. albicans* cell lysates following SDS-PAGE separation. These membranes were probed with unpurified scFv antibodies (Fig. 5A). The results revealed that IgY from the sixth immunization, cAls3-L1, and cAls3-L7 exhibited binding signals to both purified Als3 protein and *C. albicans* cell lysates, whereas cAls3-S1 showed no detectable binding signals. Additionally, a cell-based ELISA was performed to further assess binding activity. *C. albicans* cells were fixed in 96-well plates, and purified IgY antibodies from pre- and post-immunization, as well as purified scFv antibodies, were applied in serial dilutions as probes (Fig. 5B). IgY from pre-immunization displayed no binding signals, while IgY from the sixth immunization demonstrated specific binding, with signal intensity diminishing as dilution increased. Similarly, all three purified scFv antibodies bound to *C. albicans* cells, with signal intensities decreasing with higher dilutions. Among the scFv antibodies, cAls3-L7 exhibited the strongest binding activity, maintaining an OD value greater than 1.0 at a concentration of 6.25 µg/mL, compared to cAls3-S1 and cAls3-L1, which showed similar binding activities at a higher concentration of 25 µg/mL.

## Discussion

*C. albicans*, a commensal organism within the human microbiota, is a major cause of systemic fungal infections, particularly in immunocompromised patients and critically ill individuals in hospital settings (Tong and Tang 2017). The increasing incidence of *C. albicans*-associated bloodstream infections presents a significant global healthcare challenge due to their high morbidity and mortality rates. Over recent decades, substantial efforts, including ongoing clinical trials, have been devoted to developing effective strategies to combat *Candida* infections (Sahu et al. 2022). Biofilm formation not only enables *C. albicans* to achieve high cellular densities but also facilitates the exchange of genetic material, contributing to increased tolerance and resistance to antimicrobial agents (Hall and Mah 2017). Furthermore, Als3, a member of the Als family, plays a pivotal role in mediating fungal-bacterial interactions between *C. albicans* and pathogens such as *Staphylococcus aureus*, *Porphyromonas gingivalis*, and *Streptococcus gordonii* (Sztukowska et al. 2018). Importantly, prior studies have correlated *ALS* gene expression levels with fluconazole resistance, highlighting their involvement in antifungal resistance mechanisms (Roudbarmohammadi et al. 2016). As a result, the Als3 protein has emerged as a promising target for the development of novel diagnostic and therapeutic strategies against *C. albicans*. Antibody-based approaches targeting Als3 present a particularly attractive avenue, as they hold the potential to inhibit biofilm formation, disrupt fungal-bacterial interactions, and overcome antifungal resistance.

The Als3 protein was expressed and purified from a yeast expression system (Fig. 1A). The yeast expression system was selected for its ability to closely replicate the native form of Als3 in *C. albicans*, given the similarities between yeast family members. This approach ensures that the purified protein maintains structural and functional properties akin to its native state. In developing antibodies, cost-effectiveness in the purification process is a critical consideration (Dias da Silva and Tambourgi 2010). Chickens were chosen as the immune host in this study due to their numerous advantages in antibody production, particularly the ease of collecting IgY antibodies from eggs. This method is practical, economical, and noninvasive, as eggs can be collected daily, and IgY antibodies can be purified without requiring invasive procedures such as bleeding. Furthermore, IgY antibodies offer unique benefits, including their lack of interaction with rheumatoid factors and the complement system, which reduces the risk of adverse effects in therapeutic applications (Carlander and Larsson 2001). The potential of IgY antibodies has been well-documented in both clinical and experimental settings, including their utility in addressing antibiotic resistance (El-Kafrawy et al. 2023; Wang et al. 2023). Chickens are highly efficient at generating robust humoral antibody responses with small antigen quantities, producing approximately 100–150 mg of IgY antibodies per egg, of which 2–10% are typically antigen-specific (Mine and Kovacs-Nolan 2002). Consistent with these findings, our study demonstrated that the immune response, as measured by purified IgY antibodies, was modestly induced following the second immunization and exhibited strong binding signals from the fourth to the sixth immunizations (Fig. 1C–D). Notably, chickens proved to be a cost-effective host for antibody production, requiring only ~ 0.3 mg of antigen over 6 weeks of immunization to elicit a robust and sustained antibody response (Fig. 1B). This efficiency underscores the practicality and viability of using chickens in large-scale antibody production for both diagnostic and therapeutic applications.

Recombinant monoclonal scFv antibodies have been widely utilized to target a diverse array of tumor-associated antigens and infectious diseases (Munoz-Lopez et al. 2022; Roth et al. 2021). Compared to traditional hybridoma technology, recombinant antibodies offer significant advantages, including reduced cost, improved time efficiency, and adaptability to various applications. Among the available platforms, the chicken scFv system stands out for its simplicity and efficiency, requiring only a single pair of primers to generate antibody libraries (Andris-Widhopf et al. 2000). In this study, two scFv antibody libraries were constructed from immunized chickens using PCR with either 7- or 18-amino-acid peptide linkers, resulting in library sizes of 5.3 × 10^7^ and 2.8 × 10^7^ transformants, respectively. The use of hyperimmune chickens as the source of immunoglobulin cDNA significantly reduced the library size needed to generate highly specific antibodies, compared to naïve libraries that typically require much greater diversity (Maynard and Georgiou 2000). Specific binding responses were observed after only three rounds of bio-panning, as confirmed through phage-based ELISA (Fig. 2B). This process led to the identification of three distinct scFv antibodies targeting the Als3 protein (Fig. 3, Table 1). The superior efficiency of immunized libraries was evident, as they facilitated faster and more efficient selection of specific antibodies compared to naïve libraries, which often require four to six or more rounds of bio-panning (Gao et al. 2002). Furthermore, the entire bio-panning and scFv binding confirmation process was completed within 2–3 weeks, significantly shorter than the 1–2 months typically required for hybridoma technology (Pandey 2010). These findings underscore the advantages of phage display technology over hybridoma methods for the rapid selection of specific antibodies in chickens. However, the promiscuous pairing of *V*_L_ and *V*_H_ chain fragments in the *E. coli* expression system may introduce inconsistencies, which warrants further investigation (Hammers and Stanley 2014). To address potential concerns, we propose referring to these *E. coli*-derived anti-Als3 scFv molecules as “antibody-like molecules” or “recombinant Als3-binding proteins” to avoid misinterpretation. Despite these challenges, the primary goal of generating molecules with binding activity against the Als3 protein was successfully achieved, demonstrating the feasibility and efficiency of this approach.

Sequence analysis of the constructed libraries revealed two distinct groups within each library. Interestingly, the cAls3-S3 group consisted of three identical clones that lacked binding activity in the ELISA assay (Table 1, Fig. 3), a result consistent with findings from previous studies (Lee et al. 2022). Despite being successfully amplified and selected, these clones exhibited no functional specificity toward the Als3 protein. Such non-binding clones could arise due to limited library diversity, variations in antibody-antigen interactions during selection, or structural differences between antibody expression forms in the phage display and *E. coli* systems, which may affect antigen-binding activity. However, the precise cause remains unclear, emphasizing the need for further studies to investigate the structural and functional consistency of recombinant antibodies across expression platforms. Antibody diversity is generated through mechanisms such as somatic hypermutation, *V-J* gene recombination (with additional D regions for *V*_H_), and the random pairing of *V*_H_ and *V*_L_ chain variable regions in vertebrates. Of these, the CDRs, particularly CDR3, are the most variable and play a pivotal role in antigen recognition (Xu and Davis 2000). In this study, sequence analysis revealed high mutation rates in the CDR regions of the selected scFv antibodies, especially in *V*_H_ CDR3, compared to FRs and chicken germline sequences (Table 2) (Xu and Davis 2000). These elevated mutation rates strongly suggest that the selected scFvs were derived from Als3 antigen-induced immune responses rather than naïve IgM sequences. This highlights the efficacy of the system in generating highly specific antibodies capable of precise target antigen recognition.

The powerful application of antibodies lies in their capacity to function as diagnostic and therapeutic biomarkers, with binding affinity serving as a critical indicator of their potential and effectiveness. To evaluate the affinities of the selected clones, we expressed and purified three scFv antibodies using Ni^2^⁺ Sepharose, achieving results consistent with prior studies (Fig. 4A) (Lee et al. 2022). During this process, we observed a discrepancy in the apparent molecular weights of cAls3-L1 and cAls3-L7, despite their mRNA sequences differing by only 15 nucleotides. A similar phenomenon was sometimes observed in the characterization of other specific scFv antibodies against various proteins in our laboratory. While the exact cause remains unclear, possible contributing factors include differences in post-translational modifications, protein folding within the *E. coli* expression system, or SDS-PAGE migration anomalies due to charge variations. Direct ELISA assays revealed varying binding limits among the antibodies, ranging from 78 ng/mL to 20 ng/mL, reflecting strong binding activities (Fig. 4B). Competitive ELISA further confirmed these findings, and Kd values were determined using the Klotz plot method. Specifically, the Kd values for cAls3-S1, cAls3-L1, and cAls3-L7 were calculated as 4.5 × 10^−8^ M, 1.5 × 10^−8^ M, and 6.8 × 10^−8^ M, respectively (Fig. 4C). For a more accurate determination of Kd values, advanced methods such as surface plasmon resonance (SPR) are recommended. SPR could provide precise binding affinity measurements, enabling a more reliable assessment. Additionally, based on prior experience, scFv antibody binding affinities can be enhanced by at least tenfold through fusion with the Fc receptor to generate scFv-Fc fusion antibodies. In this study, the selected scFv antibodies demonstrated the ability to recognize endogenous Als3 protein in *C. albicans*, showing varying degrees of binding activity (Fig. 5). Als3 protein plays a significant role in *C. albicans* attachment and biofilm formation (Roudbarmohammadi et al. 2016; Zhao et al. 2004). Western blot analysis revealed relatively weak signals, likely due to low Als3 expression levels in *C. albicans* cultured overnight without stimulation. However, culturing *C. albicans* under CO₂-enriched conditions in 96-well plates promoted cellular attachment, potentially upregulating Als3 expression and enhancing antibody binding. Interestingly, cAls3-S1 exhibited binding activity to *C. albicans* in the ELISA assay but showed no detectable signals on western blot (Fig. 5). This discrepancy suggests that cAls3-S1 might recognize a conformational epitope distinct from those targeted by cAls3-L1 and cAls3-L7, underscoring differences in epitope specificity. Notably, the anti-Als3 scFv antibodies displayed superior binding affinity compared to previously developed antibodies targeting *Streptococcus pneumoniae* α-enolase. However, variations in target protein expression levels could account for the lower binding limits observed in this study, emphasizing the need for further optimization of culture conditions and antibody validation assays.

In conclusion, we developed a cost-effective strategy leveraging chicken immunization to generate both polyclonal IgY and monoclonal scFv antibodies targeting the *C. albicans* Als3 protein. The polyclonal IgY antibodies, purified from egg yolks, demonstrated specific binding to recombinant Als3 protein. Using phage display technology, two scFv antibody libraries were constructed efficiently, yielding three scFv antibodies with specific binding to Als3, as confirmed by western blot and cell-based ELISA assays detecting endogenous Als3 in *C. albicans*. These antibodies also exhibited strong binding affinities, with Kd values in the range of ~ 10^−8^ M, underscoring their potential as diagnostic and therapeutic tools against *C. albicans* infections. Future work will focus on optimizing these scFv antibodies further, including evaluating their ability to inhibit biofilm formation and their application in sandwich ELISA for rapid detection. Additionally, the antibodies could be conjugated with therapeutic agents or fused with the Fc region to enhance their diagnostic utility and efficacy in biomedical applications such as biofilm inhibition and rapid detection assays.

## Data Availability

All data generated or analyzed during this study are included in this published article.
